# Genome Sequence of Escherichia coli Isolated from an Adult in Kibera, an Urban Informal Settlement in Nairobi, Kenya

**DOI:** 10.1128/mra.01241-21

**Published:** 2022-03-28

**Authors:** Gilbert K. Kikwai, Bonventure Juma, Fredrick Nindo, Caroline Ochieng, Newton Wamola, Kevin Mbogo, Douglas R. Call, Elizabeth Hunsperger

**Affiliations:** a Diagnostic and Laboratory System Programme, Center for Global Health Research, Kenya Medical Research Institute, Nairobi, Kenya; b Bioinformatics and Informatics Platform, Institute of Research on Cancer and Aging, Medical School, University of Cote d’Azur, Nice, France; c Division of Medical Microbiology, Department of Pathology, Faculty of Health Sciences, University of Cape Town, Cape Town, South Africa; d Division of Global Health Protection, Centers for Disease Control and Prevention-Kenya, Nairobi, Kenya; e Paul G. Allen School for Global Health, Washington State University, Pullman, Washington, USA; f Biochemistry Department, College of Health Sciences, Jomo Kenyatta University of Agriculture and Technology, Nairobi, Kenya; University of Maryland School of Medicine

## Abstract

An Escherichia coli strain (sequence type 636) was isolated from an adult residing in an urban informal settlement in Nairobi, Kenya, and was sequenced using the Illumina MiSeq platform. The draft genome was 5,075,726 bp, with a Col(BS512) plasmid plus *aph(6)-Id*, *bla*_TEM-1B_, and *dfrA7* genes, which encode kanamycin, ampicillin, and trimethoprim resistance proteins, respectively.

## ANNOUNCEMENT

Escherichia coli is a facultative anaerobic bacterium that is able to exist within a mammalian tract as a harmless commensal or as a pathogen. The bacterial genome can acquire or lose genetic information, which can offer competitive advantages for individual strains (1, 2).

We report the genome sequence of an Escherichia coli sequence type 636 (ST636) strain that was isolated from an adult residing in Kibera, an urban informal settlement in Nairobi, Kenya. This community is characterized by poor sanitation, unregulated antibiotic use, outdoor food vending, and poor water supplies (3, 4). Sample collection was performed using protocol SSC 2998 of the Scientific and Ethical Review Unit of the Kenya Medical Research Institute and protocol 14413 of the Washington State University institutional review board. The strain was isolated by spread-plating a stool sample on a MacConkey plate. After 12 to 18 h of incubation at 37°C, a single pink/rose colony was transferred to Luria-Bertani broth and stored at −20°C.

The selected E. coli isolate was revived on Trypticase soy agar for 24 h at 37°C, and genomic DNA was then extracted by using a Qiagen DNeasy blood and tissue kit. The concentration was determined by using a Qubit double-stranded DNA (dsDNA) assay kit (Invitrogen, Carlsbad, CA, USA) according to the manufacturer’s instructions. Genomic DNA libraries were prepared with a Nextera XT library preparation kit (Illumina, USA), and paired-end sequencing was completed by using a MiSeq sequencer (Illumina, Inc., San Diego, CA, USA) with 2 × 250 cycles.

Raw reads were quality filtered and subsequently trimmed with Trimmomatic v0.38 (5) using the settings sliding window: 4:15, leading: 3, and minlen: 50. *De novo* assembly was performed with SPAdes v3.13.0 using default assembly parameters (6). The contigs obtained were filtered using in-house scripts (https://doi.org/10.6084/m9.figshare.17126777) to retain those that met minimum length and coverage criteria of 500 bp and 1.5× coverage, respectively, and the quality of the assembly was assessed using QUAST v5.0.2 (7). Multilocus sequence typing (MLST), plasmid, and resistome profiles were generated using staramr v0.7.1 (8–10), and a phage profile was generated using the PHASTER 2016 update (11). The assembled genomes were annotated using NCBI PGAP v5.3 (12). Default parameters were used for all software unless otherwise specified.

A total of 1,038,300 raw paired-end reads were obtained from sequencing, with 1,021,121 reads being retained after quality trimming, and the GC content (50.52%) was as expected. *De novo* assembly yielded 480 contigs, with a combined length of 5,140,290 bases and an *N*_50_ value of 552,852 bp. After removal of contigs that were <500 bp and lacked 1.5× coverage, the remaining data set was composed of 325 contigs, with an *N*_50_ value of 552,851 bp and 100× *g*enome coverage. The total draft genome length was 5,075,726 bp.

The identified ST was ST636, and a Col(BS512) plasmid was present. Phage analysis identified three prophage regions, of which two regions were intact; these included PHAGE_Salmon_Fels_2_NC_010463 (35.9 kbp), PHAGE_Yersin_L_413C_NC_004745 (32.3 kbp), and PHAGE_Entero_lambda_NC_001416 (31.7 kbp). Annotation revealed that the ST636 strain contains 4,647 coding sequences, 4,739 putative genes, 9 rRNAs, 81 tRNAs, and 1 transfer-messenger RNA. Antimicrobial resistance genes, including *aph(6)-Id*, *bla*_TEM-1B_, and *dfrA7*, which encode proteins for resistance to kanamycin, ampicillin, and trimethoprim, respectively, were found on the chromosome.

### Data availability.

The whole-genome sequence of the Escherichia coli ST636 strain has been deposited in GenBank under accession number GCA_020005225.1, and raw reads can be found under SRA accession number SRX13373163.
